# Observation of ‘hidden’ planar defects in boron carbide nanowires and identification of their orientations

**DOI:** 10.1186/1556-276X-9-30

**Published:** 2014-01-15

**Authors:** Zhe Guan, Baobao Cao, Yang Yang, Youfei Jiang, Deyu Li, Terry T Xu

**Affiliations:** 1 Department of Mechanical Engineering and Engineering Science, The University of North Carolina at Charlotte, Charlotte, NC 28223, USA; 2 Department of Mechanical Engineering, Vanderbilt University, Nashville, TN 37235, USA

**Keywords:** Boron carbide nanowires, Rhombohedral crystal system, Transmission electron microscopy, Planar defects

## Abstract

The physical properties of nanostructures strongly depend on their structures, and planar defects in particular could significantly affect the behavior of the nanowires. In this work, planar defects (twins or stacking faults) in boron carbide nanowires are extensively studied by transmission electron microscopy (TEM). Results show that these defects can easily be invisible, i.e*.*, no presence of characteristic defect features like modulated contrast in high-resolution TEM images and streaks in diffraction patterns. The simplified reason of this invisibility is that the viewing direction during TEM examination is not parallel to the (001)-type planar defects. Due to the unique rhombohedral structure of boron carbide, planar defects are only distinctive when the viewing direction is along the axial or short diagonal directions ([100], [010], or $\left[\bar{1}10\right]$) within the (001) plane (in-zone condition). However, in most cases, these three characteristic directions are not parallel to the viewing direction when boron carbide nanowires are randomly dispersed on TEM grids. To identify fault orientations (transverse faults or axial faults) of those nanowires whose planar defects are not revealed by TEM, a new approach is developed based on the geometrical analysis between the projected preferred growth direction of a nanowire and specific diffraction spots from diffraction patterns recorded along the axial or short diagonal directions out of the (001) plane (off-zone condition). The approach greatly alleviates tedious TEM examination of the nanowire and helps to establish the reliable structure–property relations. Our study calls attention to researchers to be extremely careful when studying nanowires with potential planar defects by TEM. Understanding the true nature of planar defects is essential in tuning the properties of these nanostructures through manipulating their structures.

## Background

Planar defects, such as stacking faults and twins, naturally exist in some as-synthesized one-dimensional (1D) nanostructures [1]. In addition to assisting the growth of nanostructures [1], these defects can affect the mechanical [2], electrical [3], thermal [4], and optical [5] properties of 1D nanostructures. Thus, it is crucial to know their nature such as existence, distribution, and orientation within each 1D nanostructure while establishing the structure–property relations. So far, transmission electron microscopy (TEM) has been one major technique commonly used to characterize the structure of individual 1D nanostructures and reveal the nature of planar defects [6]. However, due to the sophistication of the TEM technique, sometimes, experimental artifacts could be erroneously interpreted or lead to controversy [6-10]. To date, most planar defect-related studies have been focused on 1D nanostructures made of silicon, silicon carbide, III-V (e.g*.*, GaAs, InP), or II-IV compounds (e.g*.*, ZnO, CdSe) whose crystal structures are either cubic or hexagonal [8-15].

Boron carbide 1D nanostructures have attracted increasing attention in the last few years because of their potential applications in nanocomposites and thermoelectric energy conversion [16-25]. Most reported boron carbide 1D nanostructures were synthesized by carbothermal reduction or chemical vapor deposition at approximately 1,100°C [16-23]. Field emission [18,23], photoluminescence [19], mechanical [21,23], and thermal conductivity [22] properties of these 1D nanostructures were reported. However, due to the complicated rhombohedral crystal structure, detailed structural characterization especially on planar defects that could greatly affect the properties of boron carbide 1D nanostructures has not yet gained enough attention, and the structure–property relations have not been established. In our previous study [22], about one hundred as-synthesized boron carbide nanowires were subjected to TEM study, during which each nanowire was examined throughout the full tilting range allowed by the configuration of our microscope. Approximately 75% examined nanowires were found to have planar defects, while the remaining 25% were planar defect-free-like. The defected nanowires were further categorized into two groups: transverse faults (TF) nanowires with planar defects perpendicular to the preferred growth direction of nanowires and axial faults (AF) nanowires with planar defects parallel to the preferred growth direction of nanowires. The determination of defects’ existence and fault orientations (TF or AF) within each nanowire was based on the characteristic features presented in TEM results, including modulated contrast in high-resolution TEM (HRTEM) images and streaks in diffraction patterns.

In this work, more extensive TEM examination and model simulation were performed to gain a deeper understanding of the nature of planar defects in the aforementioned boron carbide nanowires to answer two questions. (1) Do planar defect-free boron carbide nanowires really exist? Literature review shows that due to its relatively low stacking fault energy (75 mJ/m^2^) [26], planar defects have been frequently observed in bulk boron carbides independent of the synthesis methods [27-30]. It has also been reported that the density of planar defects decreases as the synthesis temperature increases [30]. However, the planar defects were still detectable by TEM from bulk samples synthesized at 2,100°C [30]. Considering the common existence of planar defects in bulk boron carbides and the relatively low temperatures researchers used to synthesize boron carbide 1D nanostructures, one may naturally ask ‘Can boron carbide nanowires synthesized at approximately 1,100°C be planar defect-free? Or defects always exist but sometimes are not found by TEM?’ (2) If planar defects exist in all of our as-synthesized boron carbide nanowires, can their orientations be determined from TEM results showing no characteristic features (i.e*.*, results from the off-zone directions as discussed later)? It is expected that different orientations of planar defects could have distinctive effects on the properties of these nanowires, similar to that physical properties of superlattices could be very different along their in-plane and cross-plane directions [31,32]. Therefore, it is important to know the fault orientation of each boron carbide nanowire when establishing the structure–property relations.

In this paper, a thorough discussion on observing planar defects in boron carbide nanowires by TEM is presented. Results show that planar defects can be easily invisible in boron carbide nanowires even after a full range of tilting examination. Extra attention must be paid and reliable conclusion can only be made based on the results from different viewing directions (i.e*.*, zone axes). Furthermore, a new approach is developed to determine the fault orientations of those boron carbide nanowires whose planar defects are invisible in TEM results. The approach can be extended to other 1D nanostructures whose crystal structure is not rhombohedral.

## Methods

Boron carbide nanowires were synthesized by co-pyrolysis of diborane and methane over nickel-coated semiconductor substrates at relatively low temperatures in a home-built low-pressure chemical vapor deposition system [22]. The as-synthesized nanowires were first transferred from substrates to a small block of elastomeric polydimethylsiloxane (PDMS) by a gentle stamping process. Individual boron carbide nanowires were selected and picked up by a sharp probe mounted on an in-house assembled micromanipulator and then transferred to a TEM grid layered with lacy carbon support film. This operation was done under an optical microscope equipped with long working distance objective lenses. In each mesh of the TEM grid, only one nanowire was placed. During TEM study, each nanowire was subjected to a full range of tilting examination. The tilting range was set by the configuration of our microscope, as described later. For the nanowire that appeared to be planar defect-free in the initial round of TEM examination, it would be picked up by the sharp probe and repositioned onto another region of the lacy carbon support film for reexamination. This challenging and tedious reposition-reexamination process was repeated several times for some nanowires to reveal the true nature of planar defects inside them.

A JEOL JEM-2100 LaB_6_ transmission electron microscope, Akishima-shi, Japan, was used to characterize boron carbide nanowires. The microscope is equipped with an analytical high-resolution pole piece, which can realize a point resolution of 0.23 nm, a lattice resolution of 0.14 nm, and a specimen tilting range of ±30° in both *X* and *Y* directions. A JEOL double-tilt holder was used to realize the wide angle of tilting. It is worth pointing out that the 60° in total tilting range is comparable to or even wider than that of the most microscopes researchers used to study 1D nanostructures. The operation acceleration voltage used for this study was 200 kV.

Software packages CrystalMaker® and SingleCrystal™, Oxfordshire, UK, were used to construct, display, and manipulate three-dimensional models of boron carbide unit cell and nanowires, as well as to simulate corresponding electron diffraction patterns. All crystallographic indexes used in this paper are expressed in the rhombohedral notation for convenience of discussion (see Additional file 1 for conversion between the rhombohedral notation and the hexagonal notation).

## Results and discussion

### ‘Hidden’ defects

#### The existence of ‘hidden’ defects

**Figure 1:**
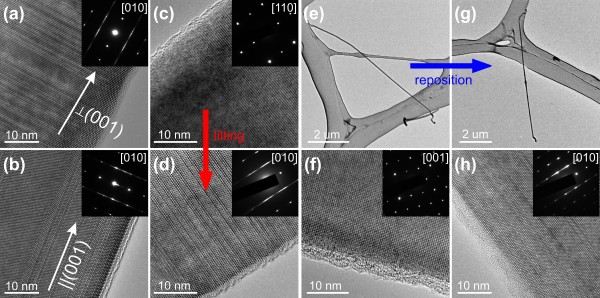
**Typical TEM results.** Results of **(a)** a TF nanowire whose preferred growth direction is perpendicular to (001) planar defects and **(b)** an AF nanowire whose preferred growth direction is parallel to (001) planar defects. Results of a nanowire whose planar defects are **(c)** invisible along the [110] zone axis, but **(d)** clearly revealed after titling to the [010] zone axis. Results of **(e)** a nanowire whose planar defects **(f)** are invisible after a full range of tilting examination. The same nanowire **(g)** was picked up and repositioned by a micromanipulator. Planar defects **(h)** are now clearly shown.

Our previous work [22] showed that {100}-type planar defects such as stacking faults and twins of variable width are commonly observed from as-synthesized boron carbide nanowires. The planar defects can be further categorized into transverse faults and axial faults, depending on the geometrical relation between the planar defects and the preferred growth direction of a nanowire. Figure 1a,b shows the typical HRTEM images of a TF nanowire with planar defects perpendicular to its preferred growth direction and an AF nanowire with planar defects parallel to its preferred growth direction, respectively.

As briefly pointed out in our previous report [22], wide angle of tilting during TEM examination is needed to reveal the existence of planar defects in as-synthesized boron carbide nanowires. Figure 1c shows the TEM results of a nanowire that seems to be planar defect-free due to the lack of modulated contrast in the image and streaks in the electron diffraction pattern. However, after tilting the nanowire to a different zone axis, all ‘hidden’ planar defects emerged as clearly shown in Figure 1d, revealing a TF nanowire. This example undoubtedly demonstrates that one cannot conclude that a nanowire is planar defect-free based on TEM results obtained from one single viewing direction. A full range of tilting examination from multiple zone axes is necessary to obtain a reliable conclusion.

As mentioned in the ‘Background’ section, although in our previous study, approximately 25% of boron carbide nanowires appear to be planar defect-free based on the full range of tilting examination, we are wondering whether these nanowires are really without any planar defects. Recently, using the reposition-reexamination process described in the ‘Methods’ section, we clarified this issue. Figure 1e is a low magnification TEM image of a boron carbide nanowire. An initial full range of tilting examination suggests that the nanowire is planar defect-free, as shown in Figure 1f. However, after repositioning the nanowire (Figure 1g) and reexamination, the ‘hidden’ planar defects are revealed in Figure 1h and the nanowire is identified as an AF nanowire. This example further demonstrates that the existence of planar defects cannot be fully revealed by observation from one single zone axis. Moreover, in specific occasions, even after a full range of tilting examination limited by the configuration of a microscope, there is still a possibility of neglecting the existence of planar defects. In our current study, twenty five planar defect-free-like nanowires were subjected to multiple rounds of reposition and reexamination, and planar defects were seen from all of them eventually. This new finding strongly suggests that planar defects exist in all of our as-synthesized boron carbide nanowires. However, these defects are not always visible from routine characterization.

#### The origin of ‘hidden’ defects

It is now clear that during TEM examination, planar defects can be easily invisible in boron carbide nanowires. Analysis indicates that the simplified reason for this invisibility is that the viewing direction is not along some specific directions parallel to planar defects.

**Figure 2:**
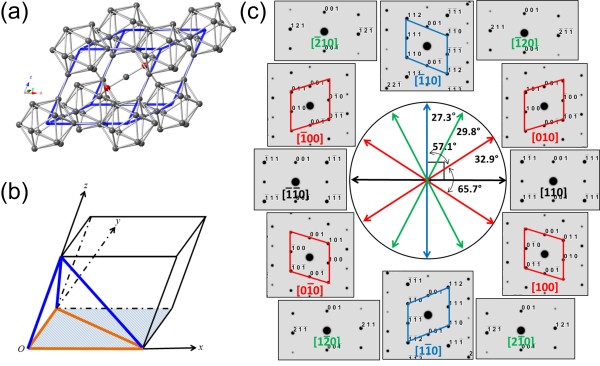
**The crystal structure of boron carbide. (a)** The rhombohedral lattice of boron carbide. Eight 12-atom icosahedra locate at the corners and one 3-atom chain occupies the longest diagonal of the rhombohedron. **(b)** A schematic drawing of the rhombohedral unit cell. The shaded plane is the (001) plane. Within the plane, orange lines represent the three in-zone directions: [100], [010], and $\;\left[\bar{1}10\right]$, along which planar defects can be observed. Blue lines represent the three off-zone directions: [001], $\;\left[10\bar{1}\right]$, and $\left[01\bar{1}\right]$, from which the planar defects cannot be seen. **(c)** A roadmap consisting of simulated diffraction patterns of major low index zone axes within the (001) plane. During TEM examination, the roadmap helps the operator to determine whether it is possible to tilt to the desired zone axes.

The crystal structure of boron carbide (Figure 2) can be viewed as a rhombohedral distortion of the cubic close packing (ccp) of B_12_ or B_11_C icosahedra [33]. The {100} planes of the rhombohedral cell are considered as the close-packed planes in the ccp arrangement. If one stacks the specific close-packed (001) plane (shaded in Figure 2b) in an ABCABC… sequence [22], a planar defect-free structure can be realized. If this normal stacking sequence is disturbed, planar defects can be formed [22] and designated as the (001)-type. During TEM examination, characteristic features of planar defects can only be seen when the viewing direction is parallel to this (001) plane. In addition, even within the (001) plane, to record TEM characteristic features of planar defects requires viewing along certain low index zone axes, which further reduces the chance of seeing the defects, as explained below.

A roadmap consisting of simulated diffraction patterns of major low index zone axes within the (001) plane is shown in Figure 2c. During TEM examination, this roadmap can help us judge if it is possible to tilt to the next zone axis according to the calculated angle between different zone axes. For example, it is nearly impossible to obtain results from both $\;\left[\bar{1}10\right]$ and [010] zone axes on the same nanowire because the calculated inter-axial angle (57.1°) is close to the tilting limit of our TEM specimen holder (60°). In the roadmap, there are four independent patterns such as those from $\;\left[\bar{1}10\right]$, $\;\left[\bar{1}20\right]$, [010], and [110] directions, as grouped in four colors. During TEM examination, planar defects can be seen along directions of $\;\left[\bar{1}10\right]$, $\;\left[\bar{1}20\right]$, and [010] whose diffraction patterns are asymmetric and with streaks in them. While viewing along the [110] direction, the layered faults feature is hidden because of the mirror symmetry. In addition, planar defects are more distinctive when viewing along directions of $\;\left[\bar{1}10\right]$ and [010] than that of $\;\left[\bar{1}20\right]$ (see Additional file 1 for comparison between experimental results obtained from the aforementioned four different zone axes). Therefore, in our real TEM practice, only results from the two independent directions: $\;\left[\bar{1}10\right]$ and [010] are recorded and analyzed.

There are a total of six equivalent $\left[\bar{1}10\right]$-type and [010]-type directions in the rhombohedral system, as drawn in orange and blue lines in Figure 2b. Characteristic features of planar defects can be observed by TEM when the viewing direction is along the rhombohedral axes or the short diagonal within the (001) plane, i.e*.*, the directions of [100], [010], and $\;\left[\bar{1}10\right]$. These three directions (outlined in orange) are denoted as in-zone directions. Meanwhile, the other three directions: [001], $\;\left[10\bar{1}\right]$, and $\left[01\bar{1}\right]$, located out of the (001) plane (marked in blue), are denoted as off-zone directions, due to the fact that planar defects are invisible from them.

Now the difficulty to visualize planar defects in boron carbide nanowires becomes obvious. If the viewing direction is not parallel to planar defects, the defects will be invisible. In addition, even if the viewing direction is parallel to planar defects, depending on the initial orientation of the viewing direction, planar defects may also not be observed. For example, if the initial viewing direction (i.e*.*, without any tilting of the specimen holder) is along the [110] direction within the (001) plane, it is then impossible to see any characteristic features of planar defects even after a full range of tilting examination. This is due to that approximately ±33° is needed to tilt from the [110] direction to the in-zone directions: [010] or [100], according to the roadmap shown in Figure 2c. This required titling angle exceeds the tilting limit of ±30° for our specimen holder.

#### Summary

In short, planar defects in boron carbide nanowires are likely hidden during TEM examination. There are only three specified in-zone directions, along which planar defects can be easily seen. The discussed difficulty of identifying ‘hidden’ planar defects in boron carbide nanowires calls attention to researchers to pay great cautions when analyzing microstructures of 1D nanomaterials with a complicated rhombohedral crystal structure. Although planar defects in boron carbide 1D nanostructures were neglected or misinterpreted in some previous publications [16,17,19,23], some research groups have realized this issue just like us. For instance, the two recent papers on α-rhombohedral boron-based nanostructures [34] and fivefold boron carbide nanowires [35] set good examples, in which abnormal weak diffraction spots were specifically studied and a serial tilting electron diffraction method was conducted to reveal cyclic and parallel twinning inside individual nanostructures. Different from these two works, our work focuses on planar defect-free-like nanowires whose experimental results are more deceptive (i.e*.*, showing no clue of defects from either TEM images or electron diffraction patterns) and presents out correct approaches to investigate these nanowires.

### Identification of fault orientations from the off-zone results

Based on the aforementioned results, we believe that planar defects exist in all of our as-synthesized boron carbide nanowires. During TEM examination, planar defects are invisible in some nanowires even after a full range of tilting examination. Additional manipulation to reposition these nanowires on TEM grids can help to meet the in-zone condition and eventually reveal the planar defects and their fault orientations (i.e*.*, AF or TF). However, this process is challenging and tedious, especially if multiple times of nanowire manipulation is needed. So without the reposition-reexamination process, is it possible to identify the fault orientation from results obtained from the off-zone directions? With the help of CrystalMaker® and SingleCrystal™, a new approach has been developed to achieve this goal.

#### Simulated cases along the three off-zone directions

**Figure 3:**
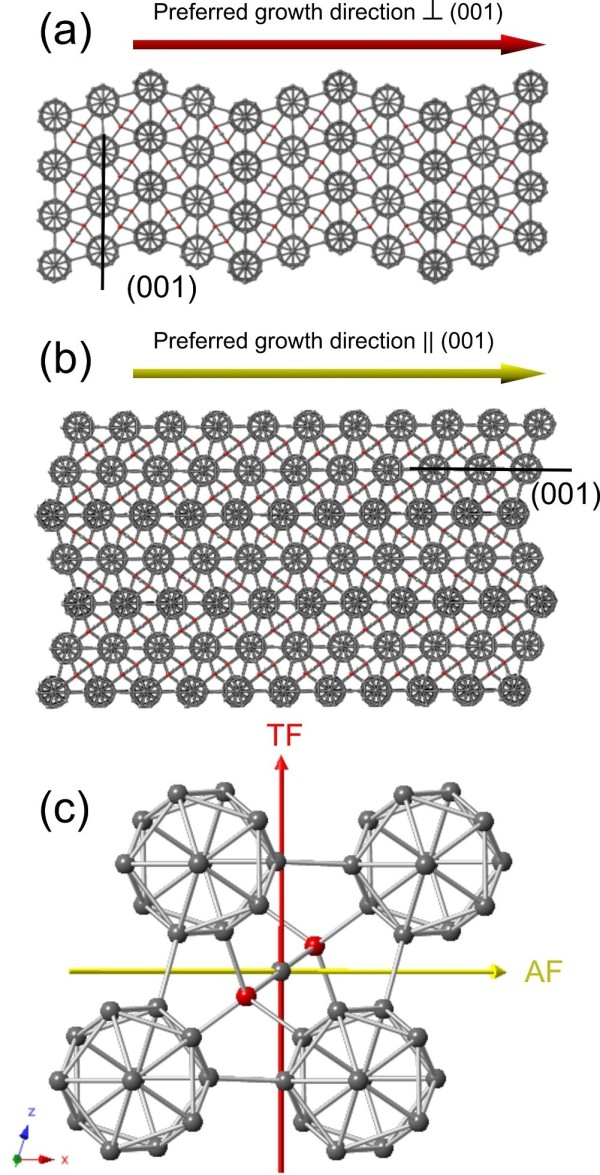
**Simulated defected nanowires labeled with corresponding projected preferred growth directions. (a)** A simulated TF nanowire whose preferred growth direction is perpendicular to (001) planes and can be indexed as $\left[\bar{0}.\bar{2}\bar{9}\bar{2},\bar{0}.\bar{2}\bar{9}\bar{2},1\right]$. **(b)** A simulated AF nanowire whose preferred growth direction is parallel to (001) planes and can be designated as [100]. **(c)** A rhombohedral boron carbide lattice viewing along the [010] direction. The aforementioned preferred growth directions are labeled on it. The red line represents the preferred growth direction of a TF nanowire, whereas the yellow line represents that of an AF nanowire.

The approach is based on the facts that (1) TF and AF nanowires have different preferred growth directions, and (2) the preferred growth direction of each type of nanowires is unique. Figure 3a is a simulated TF nanowire whose preferred growth direction is perpendicular to (001) planes. This direction can be derived geometrically as $\left[\bar{0}.\bar{2}\bar{9}\bar{2},\bar{0}.\bar{2}\bar{9}\bar{2},1\right]$. Figure 3b is a simulated AF nanowire whose preferred growth direction is parallel to (001) planes and can be experimentally determined as the [100] direction. The detailed derivation of preferred growth directions of TF and AF nanowires can be found in Additional file 1. To identify the fault orientation of a nanowire under the off-zone condition, simulation was executed on a unit cell with the aforementioned growth directions labeled on it (Figure 3c). The unit cell was tilted to the three off-zone directions, generating corresponding simulated cells and diffraction patterns. At each specific off-zone direction, for each type of nanowires, the geometrical relation between the (projected) preferred growth direction of the nanowire and diffraction spots in diffraction patterns is unique. This relation can then be used to identify the fault orientation within a nanowire whose experimental TEM data is only from the off-zone directions.

**Figure 4:**
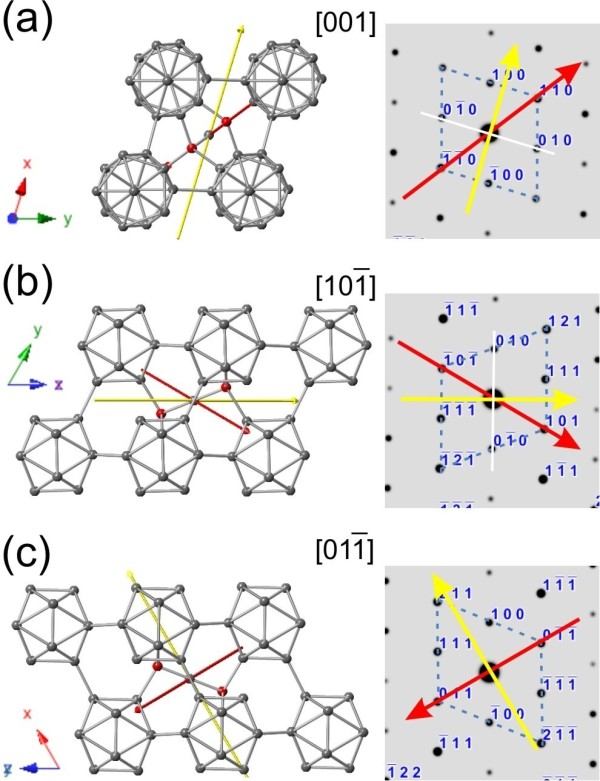
**Simulated unit cells and corresponding diffraction patterns when viewing along the three off-zone directions. (a)** [001], **(b)**$\left[10\bar{1}\right]$, and **(c)**$\left[01\bar{1}\right]$. The red and yellow lines represent the (projected) preferred growth directions of TF and AF nanowires, respectively.

Simulated unit cells and their corresponding diffraction patterns along the three off-zone directions are presented in Figure 4. The red and yellow lines indicate the (projected) preferred growth directions for TF and AF nanowires, respectively. Figure 4a is the simulated results from the off-zone [001] direction. It can be seen that the projected TF nanowire goes through $\bar{1}\bar{1}0$ and 110 spots, while the projected AF nanowire is perpendicular to the line tying the $0\bar{1}0$ and 010 spots in the diffraction pattern. These results are named as ‘TF case 1’ and ‘AF case 1’. Similarly, simulation results were obtained from the off-zone $\left[10\bar{1}\right]$ (Figure 4b) and $\left[01\bar{1}\right]$ (Figure 4c) directions, respectively. All results are further categorized into five cases, as summarized in Table 1.

**Table 1:** Simulated results for determination of fault orientation within a nanowire whose TEM results are from the off-zone directions

**Case no.**	**Zone axis**	**Alignment of the projected preferred growth direction in the diffraction pattern**
TF case 1	[001]	Through $\bar{1}\bar{1}0$ and 110 spots
TF case 2	$$\left[10\bar{1}\right]$$	Through $\bar{1}0\bar{1}$ and 101 spots
	$$\left[01\bar{1}\right]$$	Through $0\bar{1}\bar{1}$ and 011 spots
AF case 1	[001]	Perpendicular to the tie line between $0\bar{1}0$ and 010 spots
AF case 2	$$\left[10\bar{1}\right]$$	Perpendicular to the tie line between 010 and $0\bar{1}0$ spots
AF case 3	$$\left[01\bar{1}\right]$$	Perpendicular to the tie line between 011 and $0\bar{1}\bar{1}$ spots

#### Experimental validation of the simulated cases

To verify that the above simulation results indeed can be used to predict the fault orientations of boron carbide nanowires, experimental TEM data from both in-zone and off-zone conditions have to be found on the same nanowire, which turns out to be extremely challenging. It is simply impossible to achieve this goal without multiple rounds of the reposition-reexamination operation on a single nanowire, during which the nanowire could be lost or broken.

**Figure 5:**
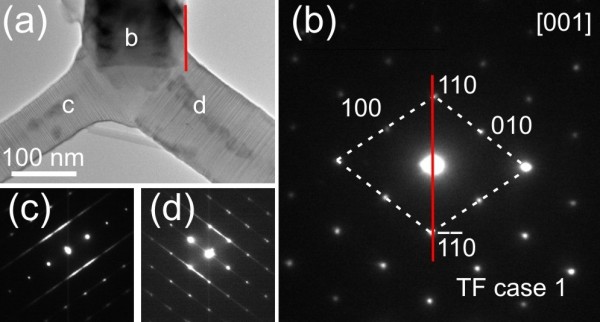
**Experimental validation of the simulated ‘TF case 1’. (a)** A boron carbide branched nanostructure made of three legs. All legs were confirmed as TF nanowires. When tilting to the [001] zone axis, **(b)** TEM results of the upper leg show no characteristic features of planar defects. However, the analyzed diffraction pattern agrees with our simulated ‘TF case 1’. TEM results of the **(c)** left and **(d)** right legs show characteristic features of TF planar defects.

For a TF nanowire, the planar defects are perpendicular to its preferred growth direction. When it is laid down on the support film of a TEM grid for examination, most of time, the viewing direction is parallel to the planar defects (see Additional file 1 for illustration). Therefore, the nanowire could be relatively easily tilted to the in-zone condition to reveal the planar defects, as the typical example shown in Figure 1c,d. In order to see the results from the off-zone directions of a TF nanowire, the nanowire has to be positioned extruding out of the support film of a TEM grid with a degree of approximately 60°, which is the angle between [001] and (001) plane, instead of laying on it. This slanting geometry is almost impossible to be realized by manipulation or tilting. So, can we still find experimental evidences to support the two simulated TF cases? Fortunately, there is a tripod-like branched structure, as shown in Figure 5, which provides solid evidence for ‘TF case 1’. For this branched structure, the three legs grew along the three rhombic planes, respectively, and all of them were confirmed to be TF nanowires (see Additional file 1 for experimental evidence). Figure 5 presents the results when the upper leg was tilted to the [001] zone axis. At this viewing direction, the left and right legs are under the in-zone condition (Figure 5a, c, d), while the upper leg is under the off-zone condition (Figure 5b). The upper leg appears to be darker because it is pointing out of the image plane. Analyzing the TEM data, the projected preferred growth direction of this leg (label as a red line) is found to go through $\bar{1}\bar{1}0$ and 110 spots, which is consistent with our simulated ‘TF case 1’.

**Figure 6:**
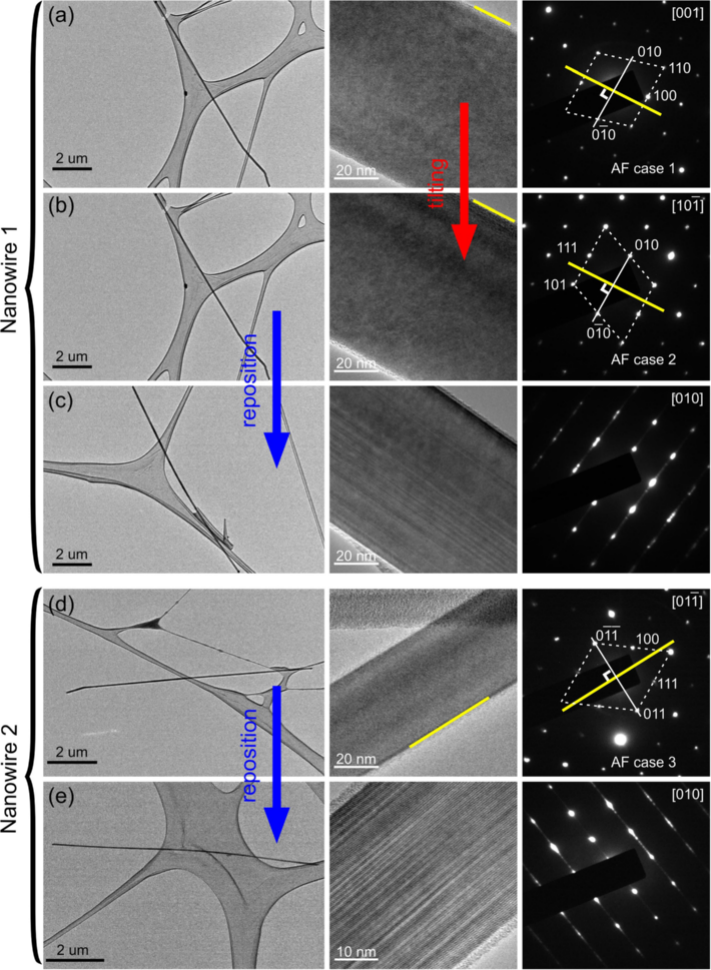
**Experimental validation of the three simulated AF cases.** TEM results of a nanowire whose planar defects are invisible from both **(a)** [001] and **(b)**$\left[10\bar{1}\right]$ zone axes. The analyzed diffraction patterns agree with the simulated ‘AF case 1’ and ‘AF case 2’, indicating that the nanowire is an AF one. **(c)** After the reposition-reexamination process, planar defects are revealed and the nanowire is confirmed to have axial faults. TEM results of another nanowire **(d, e)**, which confirm the correctness of ‘AF case 3’.

For an AF nanowire, the planar defects are parallel to its preferred growth direction. When it is randomly laid down on the support film of a TEM grid for examination, most of time, the viewing direction is not parallel to the planar defects (see Additional file 1 for illustration). In other words, results from the off-zone condition are commonly recorded and planar defects would be mostly invisible. With the help of the reposition-reexamination process, the correctness of all three simulated cases for AF nanowires was validated. Figure 6a, b, c shows the results from the same nanowire. As shown in panels a and b, the projected preferred growth directions labeled as yellow lines are perpendicular to the lines tying the 010 and $0\bar{1}0$ diffraction spots. These experimental results agree with the simulated ‘AF case 1’ and ‘AF case 2’ shown in Figure 4 and Table 1, indicating that this nanowire is an AF nanowire. After reposition, the characteristic features of planar defects are clearly revealed in Figure 6c to confirm that this nanowire is an AF one. Figure 6d, e shows the experimental results of another nanowire, which confirm the correctness of ‘AF case 3’.

#### Summary

In brief, an approach to identify the fault orientation of a nanowire based on TEM results from the off-zone condition was developed. The key of this approach is to analyze the geometrical relation between the projected preferred growth direction of a nanowire and certain diffraction spots from its diffraction patterns recorded along the off-zone directions. Comparison with experimental data shows that this approach correctly identifies the fault orientation in a boron carbide nanowire without going through the tedious reposition-reexamination process. Knowing the fault orientation of each nanowire could help us to establish reliable structure–property relations of boron carbide nanowires.

## Conclusions

In summary, a thorough discussion on the observation of planar defects in boron carbide nanowires is presented. There are two major findings. (1) Planar defects can easily become invisible during TEM examination, in which case, observation along different zone axes is a must when studying the nature of planar defects. A roadmap based on simulated diffraction patterns along several low index zone axes parallel to planar defects is constructed to facilitate the practical TEM examination. (2) An approach has been developed to determine the fault orientation (i.e., transverse faults or axial faults) within a nanowire even if the planar defects are not revealed by TEM, which could facilitate further examination of the nanowire and help to establish the structure–property relations. Although our discussion is focused on boron carbide nanowires, the above two major findings are useful when studying other 1D nanostructures. This study prompts us to use cautions when drawing the conclusion of ‘planar defect-free’ 1D nanostructures, especially for those made of materials with relatively low stacking fault energy. Last but not the least, it is worth pointing out that the current study is on long straight portions of boron carbide nanowires only. For boron carbide nanowires with kinks, new phenomena might be observed in the kinked portions, which is currently under investigation.

## Competing interests

The authors declare that they have no competing interests.

## Authors’ contributions

ZG and BC performed TEM examination and crystal model simulation. YY and YJ transferred nanowires onto TEM grids and repositioned nanowires using micromanipulators. ZG, BC, and TTX contributed to data analysis and discussion. ZG, BC, and TTX prepared the manuscript. DL and TTX supervised the project. All authors read and approved the final manuscript.

## Supplementary Material

Additional file 1 **Supplementary information on (1) conversion between rhombohedral and hexagonal notations, (2) TEM images taken from**$\left[\bar{1}10\right]$, $\left[\bar{1}20\right]$**, [010], and [110] directions, (3) determination of the preferred growth directions of TF and AF nanowires, (4) illustration of the geometrical orientations of TF and AF nanowires on TEM grids, and (5) detailed results from the tripod-like branched nanostructure.** Click here for file
