# Supplementary material for: Observation of ‘hidden’ planar defects in boron carbide nanowires and identification of their orientations
Source: Nanoscale Res Lett. 2014 Jan 15;9(1):30. doi: 10.1186/1556-276X-9-30 (PMC3898527; doi:10.1186/1556-276X-9-30)
Supplement: Additional file 1 — Supplementary information on (1) conversion between rhombohedral and hexagonal notations, (2) TEM images taken from1¯10, 1¯20, [010], and [110] directions, (3) determination of the preferred growth directions of TF and AF nanowires, (4) illustration of the geometrical orientations of TF and AF nanowires on TEM grids, and (5) detailed results from the tripod-like branched nanostructure. [file 1556-276X-9-30-S1.pdf]

Observation of "Hidden" Planar Defects in Boron Carbide Nanowires and Identification of Their Orientations

Supplementary Information

1. Conversion between Rhombohedral Notations and Hexagonal Notations

The crystallographic planes and directions in a rhombohedral unit cell can be expressed in either rhombohedral or hexagonal notations. When discussing boron carbide 1D nanostructures, both notations have been used in literatures.[1-4] According to our experience, the growth directions and the growth front planes of boron carbide nanostructures are usually the rhombohedral axes and rhombic faces. Thus, we decided to use the rhombohedral notation for convenience. The rhombohedral and hexagonal notations can be converted to each other. Assume the (*hkl*) plane and the [*uvw*] direction are for the rhombohedral system and the (*HKL*) plane and the [*UVW*] direction are for the hexagonal system, there exist the following conversion relations.[5]

$$\begin{cases} H = h - k \\ K = \quad\; k - l \\ L = h + k + l \end{cases} \qquad \begin{cases} h = (2H + K + L)/3 \\ k = (-H + K + L)/3 \\ l = (-H - 2K + L)/3 \end{cases}$$

$$\begin{cases} U = (2u - v - w)/3 \\ V = (u + v - 2w)/3 \\ W = (u + v + w)/3 \end{cases} \qquad \begin{cases} u = U + \quad\; W \\ v = -U + V + W \\ w = \quad\; -V + W \end{cases}$$

Here, for easy comparison between our presented data and other published results, a conversion table for some major planes and directions between rhombohedral and hexagonal notations is listed in Table S1.

Table S1. Conversion between Rhombohedral and Hexagonal Notations for Some Major Planes and Directions in a Rhombohedral Unit Cell

Planes		Directions	
**Rhombohedral**	**Hexagonal**	**Rhombohedral**	**Hexagonal**
$(001)_r$	$(0\bar{1}1)_h$	$[100]_r$	$\frac{1}{3}[211]_h$
$(011)_r$	$(\bar{1}02)_h$	$[010]_r$	$\frac{1}{3}[\bar{1}11]_h$
$(111)_r$	$(003)_h$	$[01\bar{1}]_r$	$[010]_h$
		$[\bar{1}10]_r$	$[\bar{1}10]_h$
		$[110]_r$	$\frac{1}{3}[122]_h$
		$[111]_r$	$[001]_h$

2. Comparison between Results from the $[\bar{1}10]$, $[\bar{1}20]$, [010] and [110] Directions

As discussed in the main paper, there are four main low index zone axes within the (001) plane, along which planar defects are initially expected to be observed from all of them. However, in reality, characteristic features of planar defects can only be easily and clearly seen from the $[\bar{1}10]$ (Figure S1(a)) and [010] (Figure S1(e)) directions, but less distinctive from the $[\bar{1}20]$ direction (Figure S1(c)), and totally invisible from the [110] direction (Figure S1(g)). The reason is that in the [110] direction, the layered faults feature is hidden because of the mirror symmetry (Figure S1(h)), while from the other three directions, each segment of the faults can be distinguished (Figure S1(b), (d), and (f)).

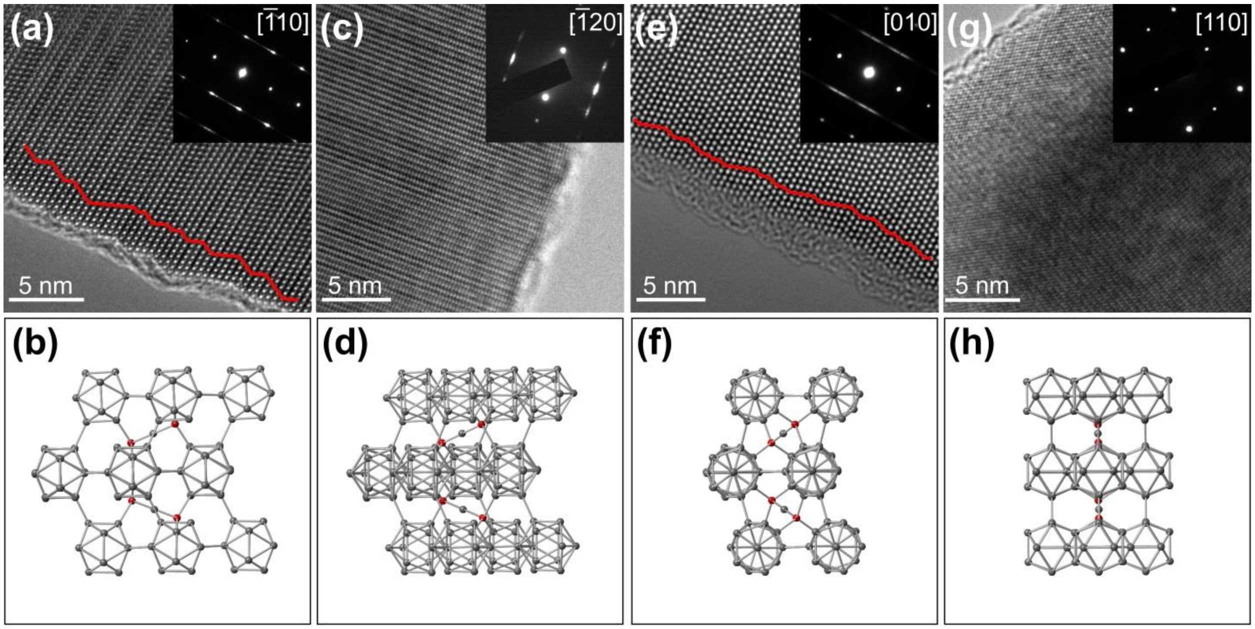

Figure S1. Observation of planar defects along the $[\bar{1}10]$, $[\bar{1}20]$, [010] and [110] directions. The four directions are all parallel to planar defects. However, planar defects are clearly visible when viewing along the direction of $[\bar{1}10]$ or [010], less distinctive along the $[\bar{1}20]$ direction, and totally invisible from the [110] direction.

3. Determination of the Preferred Growth Directions of TF and AF nanowires

Before presenting the detailed derivation of the preferred growth directions of TF and AF nanowires, two general issues are worth emphasizing.

First, TEM images and diffraction patterns from at least two zone axes are needed for determination of the preferred growth directions of nanowires. This requirement is due to the fact that the preferred growth direction shown in a TEM image is a projection of the true one. As illustrated in Figure S2, when the nanowire is not perpendicular to the incident electron beam,

what we see in the screen is the projection of the nanowire. Thus, from a single zone axis, the preferred growth direction can be any direction within the plane made of the electron beam and the projected direction (plane 1 in yellow greenish color in Figure S2). If the nanowire is tilted to the second zone axis out of the plane 1, the other plane (plane 2 in brown color) consisting of the electron beam and the nanowire projection will form. Geometrically these two planes will intersect at the line where the real nanowire is located, and the growth direction is the cross product of the Miller indexes of these two planes. This is why observations from two zone axes are necessary to determine the real preferred growth direction of a nanowire. It is needed to note that the nanowire must be tilted out of the plane 1. Otherwise, its preferred growth direction will still be unknown.

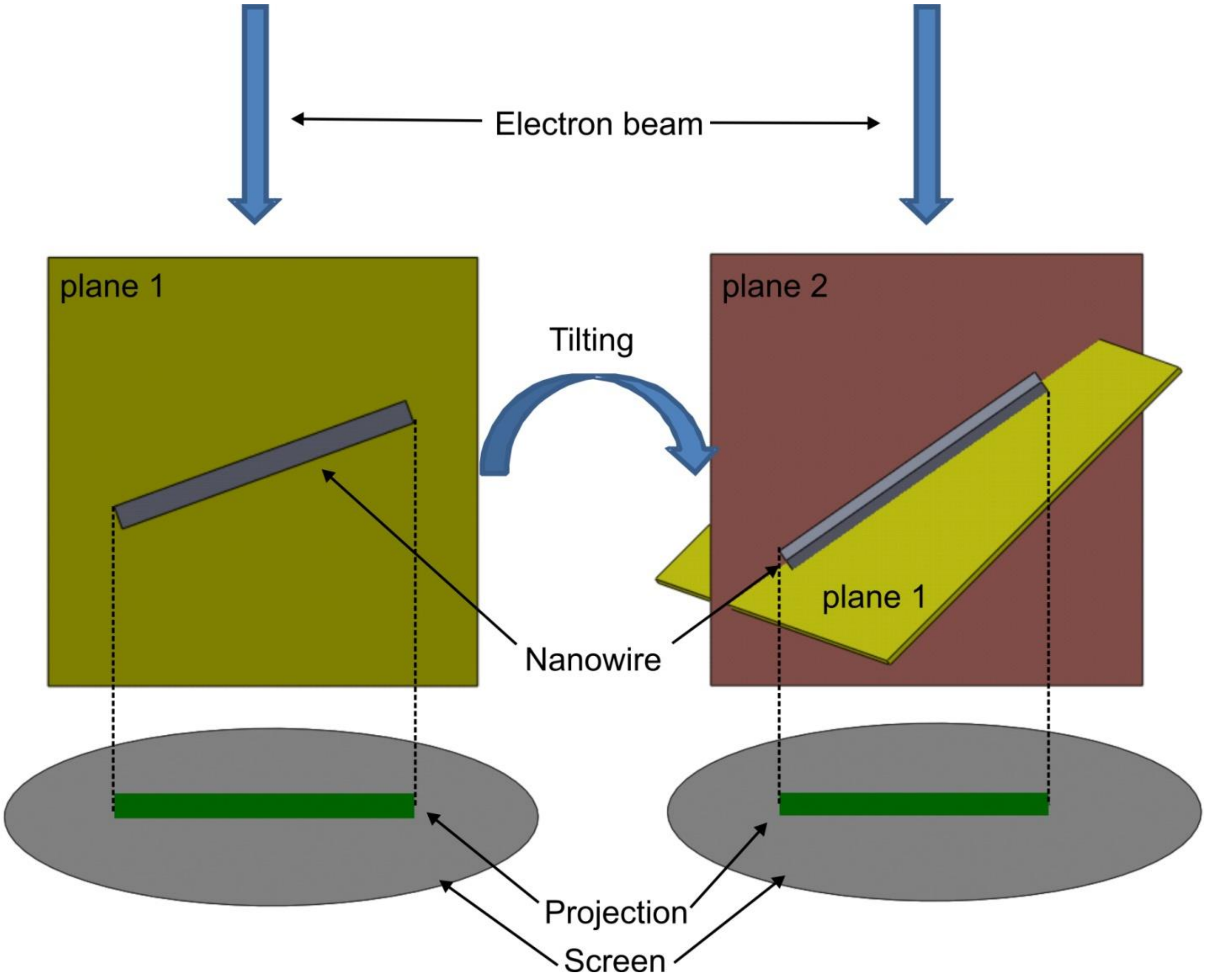

Figure S2. Illustration on why results from at least two zone axes are needed for determination of the preferred growth direction of a nanowire.

Second, people sometimes treat the index of a diffraction spot (*hkl*) as [*hkl*] and present the preferred growth direction of a nanowire is along the [*hkl*] direction. This treatment is only valid

when [*hkl*] is perpendicular to (*hkl*).[6] However for the rhombohedral crystal structure of boron carbide, in most cases, [*hkl*] is not the normal of plane (*hkl*) (*e.g.*, $[001]_r$ is not normal to $(001)_r$ as illustrated in Figure S4(a)). Therefore, when a projected boron carbide nanowire is found to go through the 001 spot in a diffraction pattern, one cannot say that [001] is the preferred growth direction. The real crystallographic indexes of the preferred growth direction should be carefully derived experimentally or geometrically, as discussed below.

3.1.The preferred growth direction of a TF nanowire

In order to use the "two-zone axes" method discussed above to determine the preferred growth direction of a nanowire, the indexes of the two planes: plane 1 and 2 in Figure S2 need to be known. Generally this goal could be achieved by the following steps. (1) Tilt the nanowire to low-index zone axes and obtain corresponding diffraction patterns. (2) Within each diffraction pattern, identify a diffraction spot that is close to the center spot and its $\vec{g}$ vector is perpendicular to the projected nanowire. (3) Index this specified diffraction spot and the obtained numbers are the crystallography indexes of the plane. Once the indexes of the two planes are known, the preferred growth direction of the nanowire can be determined.

Occasionally the two planes are hard to be expressed by low-index diffraction spots, like the case of TF nanowires in our work. As shown in Figure S3, the projected nanowire passes the (001) diffraction spots, while no diffraction spots whose $\vec{g}$ vectors are perpendicular to the projected nanowire can be easily identified. Since plane 1 and plane 2 in Figure S2 cannot be indexed here for this TF nanowire, a different procedure has to be used to obtain the real crystallography indexes of the preferred growth direction.

The procedure started with tilting the TF nanowire around the normal direction of (001) planes while paying attention to its projection image. The projection remains still only if the nanowire is tilted around its preferred growth direction. When the TF nanowire was tilted to different zone axes ($[\bar{1}10]$ and [010], Figure S3(a) and (b)) within the (001) plane, it was found that the projected preferred growth directions are always normal to the (001) plane, and at the same time, the nanowire orientation keeps the same. This observation indicates that this TF nanowire grows right along the normal direction of the (001) plane.

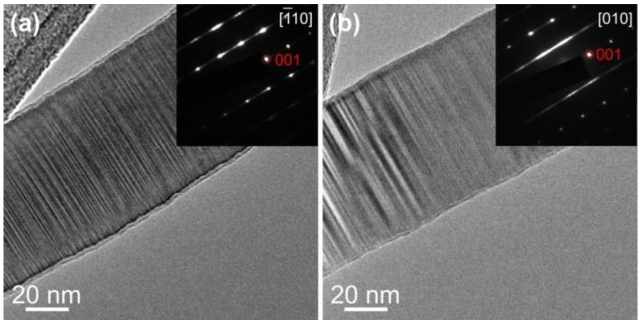

Figure S3. A TF nanowire viewed from both [$\bar{1}10$] and [010] directions. The projected preferred growth direction is always normal to the (001) plane. The real preferred growth direction can be geometrically determined as [$\overline{0.292}, \overline{0.292}, 1$], as discussed in the text.

The procedure continued as finding out the crystallographic indexes of the normal direction of the (001) plane by a geometry-based method. Figure S4(a) is a schematic drawing of a rhombohedral unit cell with some dotted lines added to facilitate the derivation. The shaded plane *OEDF* is the bottom (001) plane, within which *OD* and *EF* are the diagonals. *AC* is parallel with the normal direction of the (001) plane. It intersects *OD* at point *C*. *AB* is perpendicular to *OE*, so is *CB*. According to this geometry, we have

$$\begin{cases} |OF| = |OE| = a \\ \angle FOE = \alpha;\ \angle COB = \alpha/2 \\ \angle ABO = \angle ACO = \angle CBO = \angle OGE = 90° \end{cases},$$

where a and α are the lattice parameters of a rhombohedral unit cell. For boron carbide, α is 65.7° according to JCPDS 04-007-1018.[7]

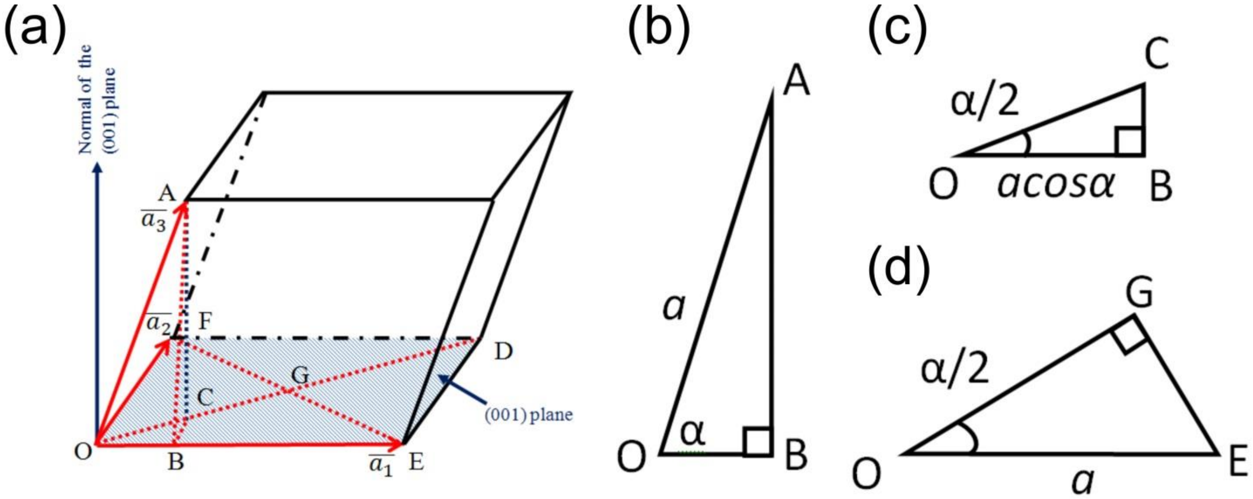

Figure S4. (a) Schematic drawing of a rhombohedral unit cell. The colored lines are added to facilitate the derivation of the crystallographic indexes of the normal of the (001) plane. (b)-(d) Useful triangles isolated from the unit cell for the geometrical derivation.

Now the task is to represent $\overrightarrow{CA}$ in terms of three unit vectors $\overrightarrow{a_1}$, $\overrightarrow{a_2}$ and $\overrightarrow{a_3}$ so that the crystallographic indexes of the normal direction of the (001) plane can be extracted.

According to vector geometry,

$$\overrightarrow{CA} = \overrightarrow{OA} - \overrightarrow{OC}$$

Since $\overrightarrow{OA}$ is the unit vector, $\overrightarrow{OC}$ is what we need to solve for $\overrightarrow{CA}$.

In the triangle AOB (Figure S4(b)),

$$|OA| = a, \angle ABO = 90°$$

So

$$|OB| = acos\alpha, |AB| = asin\alpha$$

In the triangle COB (Figure S4(c)),

$$|OB| = acos\alpha, \angle OBC = 90°$$

Therefore,

$$|OC| = \frac{acos\alpha}{cos\frac{\alpha}{2}}$$

Now we know the length of *OC*, thus the corresponding vector can be written as

$$\overrightarrow{OC} = \frac{|\overrightarrow{OC}|}{|\overrightarrow{OD}|} \cdot (\overrightarrow{a_1} + \overrightarrow{a_2}) = \frac{|\overrightarrow{OC}|}{2|\overrightarrow{OG}|} \cdot (\overrightarrow{a_1} + \overrightarrow{a_2})$$

In the triangle OGE (Figure S4(d)),

$$|OG| = a\,cos\frac{\alpha}{2}$$

Then $\overrightarrow{OC}$ can be represented by unit vectors

$$\overrightarrow{OC} = \frac{cos\,\alpha}{2\,cos^2\frac{\alpha}{2}} \cdot (\overrightarrow{a_1} + \overrightarrow{a_2})$$

$$\overrightarrow{CA} = \overrightarrow{OA} - \overrightarrow{OC} = \overrightarrow{a_3} - \frac{cos\,\alpha}{2\,cos^2\frac{\alpha}{2}} \cdot (\overrightarrow{a_1} + \overrightarrow{a_2})$$

So the normal direction is

$$[-\frac{\cos\alpha}{2\cos^2\frac{\alpha}{2}}, -\frac{\cos\alpha}{2\cos^2\frac{\alpha}{2}}, 1]$$

Taking $\alpha = 65.7°$, the crystallographic indexes of the normal direction of the (001) plane is thus determined as

$$[\overline{0.292}, \overline{0.292}, 1]$$

3.2.The preferred growth direction of an AF nanowire

For AF nanowires examined in this work, their preferred growth directions are always parallel to the (001) plane. Based on the above “two-zone axes” method, the crystallographic indexes of the preferred growth direction of AF nanowires are found to be unique and can be determined as [100].

Figure S5 shows experimental results from two different zone axes [010] and [001] of one same AF nanowire. In Figure S5(a), the projected preferred growth direction is found to be parallel to the (001) plane (like plane 1 in Figure S2). In Figure S5(b), the projected growth direction is parallel to the (010) plane (like plane 2 in Figure S2). According to the “two-zone axes” method, the real growth direction of this AF nanowire is the intersection of (010) and (001) planes, which is easy to be calculated as the [100] direction.

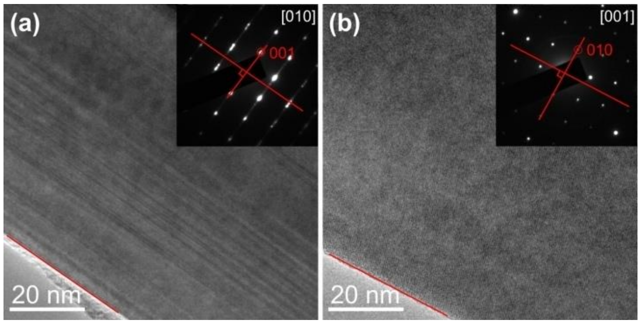

Figure S5. An AF nanowire viewed from both [010] and [001] directions. Its preferred growth direction can be experimentally determined as the [100] direction.

4. Illustration of the Geometrical Orientations of TF and AF Nanowires on TEM grids

In practical observation, “in-zone” directions generally are easier to be reached on TF nanowires than AF ones, due to the fact that planar defects are in different orientations inside these nanowires. For TF wires, planar defects are always perpendicular to the preferred growth directions (*i.e.*, axial directions). When a TF nanowire is randomly positioned on the flat support film of a TEM grid, in most cases, its planar defects would be nearly parallel to the incident electron beam, as shown in Figure S6(a). As a result, by only tilting the nanowire with respect to its axial direction, “in-zone” directions can be relatively easily reached. However, for the case of AF nanowires, planar defects are along their axial directions. When an AF nanowire is put on a TEM grid randomly, there are a few chances that the planar defects are right parallel to the

electron beam. One extreme example is shown in Figure S6(b), in which the electron beam is normal to the planar defects. Thus, to reach the "in-zone" directions of an AF nanowire, two steps are necessary. First, have the (001) plane parallel to the electron beam by tilting the nanowire with respect to its axial direction. Second, tilt within the (001) plane to reach the "in-zone" directions. As a result, it is relatively difficult and tedious to reach the "in-zone" directions of AF nanowires than that of TF nanowires.

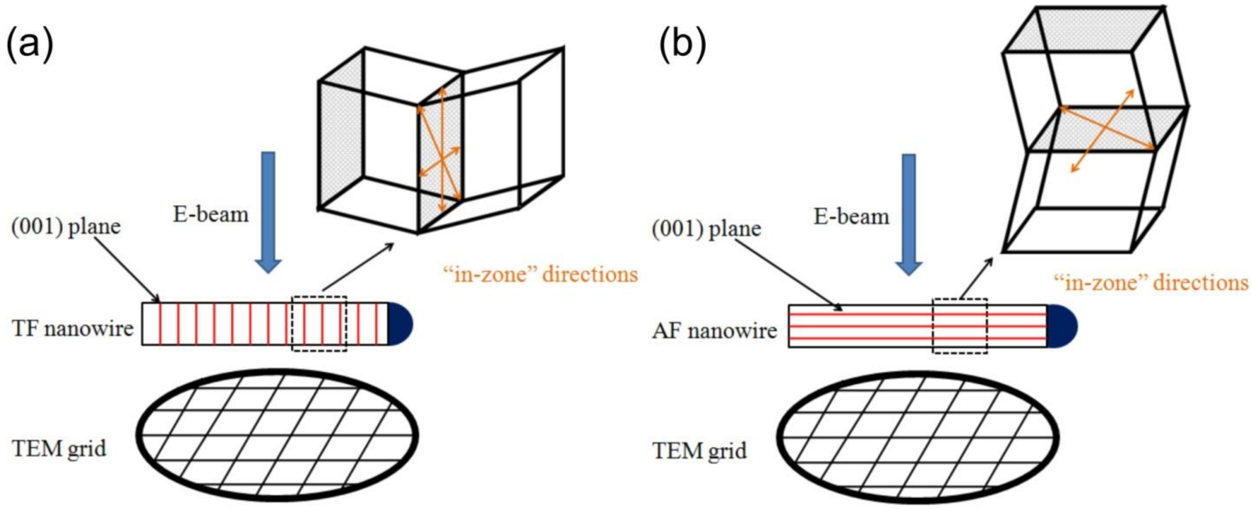

Figure S6. The relation between the incident electron beam and TF and AF nanowire positions on TEM grids

5. Detailed Results from the Tripod-like Branched Nanostructure

The tripod-like branched nanostructure discussed in the main paper has transverse faults in all three legs. For the left and right legs, the characteristic features of planar defects were observed, as clearly shown in Figures 5(b) and (c) of the main paper. For the upper leg, no characteristic features were revealed even after a full range of tilting examination. However, a new feature so-called "fused rings" (Figure S7(a)) was discovered during the titling observation. This new feature has been proved as another indicator of transverse faults.

Figures S7(b) and (c) are TEM results from one nanowire, demonstrating why "fused rings" is an indicator of transverse faults. Figure S7(b) presents results recorded from the $[\bar{1}12]$ direction, from which "fused rings" are shown in the image while no other characteristic features of planar defects can be seen. The nanowire was then tilted to the [010] "in-zone" direction as shown in Figure S7(c), from which the transverse faults are clearly revealed. Based on these results, it is reasonable to say that "fused rings" is one kind of indicators for transverse faults.

Therefore, the upper leg of the tripod-like nanostructure is a TF nanowire. More detailed structural analysis of this branched structure can be found in our recent extended abstract for the M&M 2013 conference.[8]

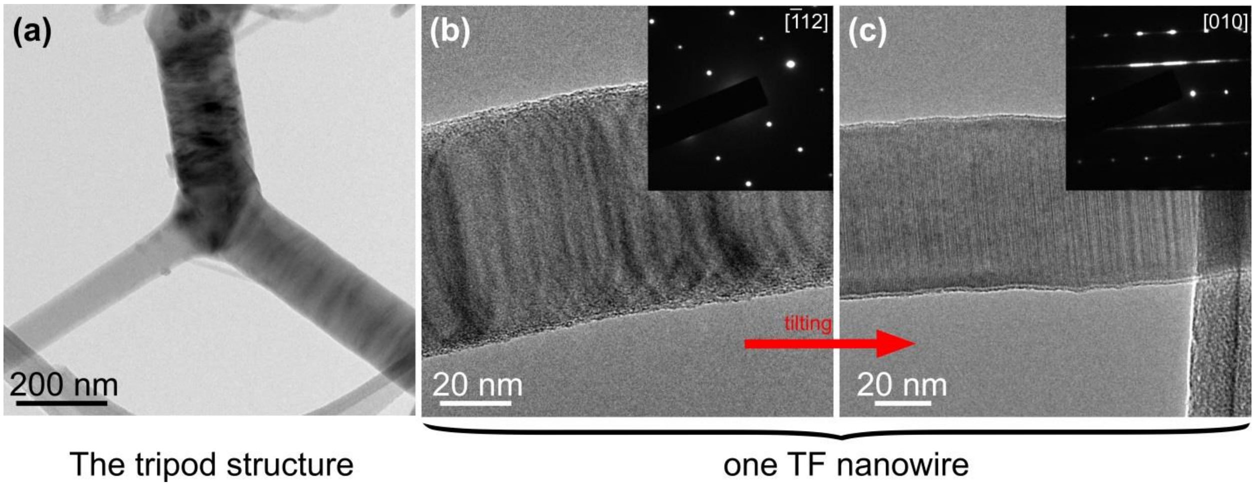

Figure S7. (a) A low magnification TEM image of the tripod-like branched nanostructure discussed in the main paper. At this viewing direction, “fused rings” are observed from the upper and right legs, whereas no special features are seen from the left leg. When viewing from other directions, the characteristic features of planar defects are shown in the left and right legs, as presented in the main paper. Evidence on why “fused rings” is an indicator of transverse faults is presented in (b) and (c). One nanowire originally showing “fused rings” when viewed along the [$\bar{1}12$] direction (b) was tilted to the [010] direction (c), from which the transverse faults are clearly revealed.

References

1. M. Carlsson, F.J. Garcia-Garcia, and M. Johnsson, *Journal of Crystal Growth*, 2002, **236**, 466-476.
2. R. Ma and Y. Bando, *Chemistry of Materials*, 2002, **14**, 4403-4407.
3. L.H. Bao, et al., *Chinese Physics B*, 2008, **17**, 4585-4591.
4. X. Fu and J. Yuan, *Nanoscale*, 2013, **5**, 9067-72.
5. W.H. Baur, *Crystal Structures I. Patterns and Symmetry*, WILEY-VCH Verlag GmbH, Washington, D. C., 1997
6. Y. Ding and Z.L. Wang, *Journal of Physical Chemistry B*, 2004, **108**, 12280-12291.
7. JCPDS 04-007-1018.
8. B. Cao, Z. Guan, and T. Xu, *Microsc. Microanal.*, 2013, **19**, 1848-1849.
